# The impact of market-incentive environmental regulation policies on corporate environmental costs: Evidence from China’s carbon trading policy

**DOI:** 10.1371/journal.pone.0297003

**Published:** 2024-02-09

**Authors:** Zhilong Qin, Chao Tu, Weihui Han, Qintong Jiang

**Affiliations:** 1 Institute of Western China Economic Research, Southwestern University of Finance and Economics, Chengdu, China; 2 Research Institute of Economics and Management, Southwestern University of Finance and Economics, Chengdu, China; 3 Business School, Beijing Normal University, Beijing, China; Second Xiangya Hospital, Central South University, CHINA

## Abstract

As the world’s largest emitter of carbon, China has implemented a series of environmental regulatory policies to reduce emissions. However, most of these environmental regulations have been at the expense of increased corporate environmental costs. Therefore, research on how to efficiently control these costs is of significant practical importance. This paper uses the China’s carbon trading policy (CTP) implemented in 2013 as a quasi-natural experiment, utilizing data from Chinese listed manufacturing firms between 2008 and 2020. Employing a difference-in-differences (DID) model, the study investigates the impact of market-incentive environmental regulatory policies (ERP) on environmental costs. The findings reveal that CTP significantly reduced the environmental costs of firms, confirming the positive and vital role market-incentive ERP can play in environmental protection and cost control. These conclusions remain robust after a series of stability tests. Mechanism analysis suggests that the cost reductions brought by market-incentive ERP are primarily achieved through increasing green innovation. Heterogeneity analysis shows that non-state-owned enterprises (non-SOEs), key polluting firms, firms with lower financial constraints, and firms with lower total production efficiency benefit more from market-incentive environmental regulatory policies. This study provides new empirical evidence for government policy-making aimed at achieving long-term sustainable development.

## 1. Introduction

Since the reform and opening up in 1978, China’s economy has rapidly developed through a high-growth model, but this extensive development has also led to a series of resource and environmental issues. In September 2020, Chinese leader announced the goals of peak carbon emissions and carbon neutrality at the 75th United Nations General Assembly. Furthermore, in the report of the 20th National Congress of the Communist Party of China, Chinese leader emphasized the need to accelerate the green transformation of development modes and actively and prudently advance towards carbon dioxide emissions peak and carbon neutrality, aiming to promote green development. As the main actors in energy saving and emission reduction, enterprises should actively respond to the national initiative of promoting green development and comply with China’s environmental regulatory policies.

In the context of addressing the global energy crisis and the increasingly severe challenges of climate change, the United Nations and its partners outlined zero-carbon emission goals in May 2020, emphasizing the urgency and significance of proactive measures in global climate governance [1]. China sees itself as an important player in this agenda too, and declared its climate policy and timeline in achieving carbon peak and carbon neutrality in September 2020. Given the pressing nature of current global climate change and the sustainable development agenda, in-depth research into Environmental Regulation Policies (ERP) within key sectors such as manufacturing has become particularly critical. In response, governments around the world have successively established relevant environmental regulations to impose environmental protection requirements on enterprises [2]. In China, manufacturing is a major carbon emitter, and also expected to take the most responsibility in carbon emission reduction. In recent years, enterprises, especially those in the manufacturing industry, have been increasingly required to take responsibility for environmental pollution [3–6], reflected by the numerous environmental regulatory policies enacted recently. These environmental regulations have raised the minimum standards for enterprise environmental performance, but often also increased their operating costs [7, 8]. Enterprises that fail to meet environmental standards may be subject to government penalties. Companies must increase their environmental expenditure to meet environmental standards, putting pressure on their financial performance. Therefore, economic performance must also be considered when analyzing the environmental performance of ERP.

While the environmental effects have been extensively studied, the impact of ERP on corporate economic performance remains a significant research gap in both academia and policy-making. This study aims to bridge this gap by analyzing the effects of China’s carbon trading policy (CTP), providing empirical evidence for balancing environmental protection and economic development. This is not only vital for policymakers to formulate more effective environmental strategies but also offers a new perspective on how enterprises respond to environmental challenges amidst rapid industrialization.

ERP is an effective approach to solving externalities caused by environmental pollution. It consists order-control ERP and market-incentive ERP [9]. Order-controlled ERP enforces the behavior of polluters through legal and administrative means. It is easy to implement and has an immediate effect, but it puts a heavy environmental burden on enterprises [10]. Realizing the "double dividend" of enterprise environmental protection and economic benefits is critical to ensuring high-quality economic development. Fortunately, market-incentive ERP provides economic instruments to adjust polluters’ costs and profits, enabling companies to balance economic costs with environmental protection. The Porter hypothesis suggests that implementing ERP can guide and encourage enterprises to find non-efficiency of resource allocation problems and potential innovation opportunities, promoting technological and organizational innovation [11]. Technological innovation can help compensate for the regulation cost and even improve corporate economic performance.

The research on ERP is divided into environmental and economic effects. This paper focuses on the economic consequences of ERP. According to previous studies, the impact of ERP on economic development could be more consistent. Some studies suggest mandatory order-controlled ERP increases corporate environmental protection expenditure, and lowers corporate business performance [12]. Other studies have shown that, under the cost constraints of market-incentive ERP, companies generally seek to reduce their costs to alleviate the economic pressure of their own environmental pollution [13, 14]. Corporate environmental cost refers to the expenses incurred by enterprises to achieve environmental protection and compliance requirements. These costs typically involve improving the environmental performance of production processes and products, reducing pollution and emissions, and meeting environmental standards and regulations set by government and regulatory authorities.

CTP is a typical market-incentive ERP, allowing enterprises to trade carbon emission rights flexibly in the market, influencing corporate decisions on carbon emissions. In 2013, China commenced pilot projects for a carbon emissions trading system. After four years of pilot exploration of carbon emission trading, the national carbon emission trading market (power industry) was officially launched in December 2017, which would be the world’s largest emissions trading system. Enterprises can obtain carbon emission rights through green innovation to reduce their fixed environmental protection expenditure while meeting environmental requirements, which is expected to impact corporate economic performance significantly. Therefore, what impacts will market-incentive ERP have on the environmental protection cost of enterprises? Is market-incentive ERP a burden for enterprises? To address this practical issue, this paper systematically analyzes the impact of market-incentive environmental regulation on corporate environmental costs, with the aim of exploring pathways for enterprises to achieve high-quality development through environmental protection and cost reduction efficiencies.

This study employs the carbon emissions trading policy implemented in 2013 as a quasi-natural experiment. Utilizing data from Chinese listed manufacturing firms between 2008 and 2020, we apply a difference-in-differences (DID) model to investigate the impact of market-incentive environmental regulatory policies on environmental costs. One possible mechanism is that enterprises can profit from eco-innovation by increasing green innovation when facing the incentives from the carbon emission trading market, thus bringing a reduction of the fixed cost of environmental protection. After a series of robustness tests, such as considering the implementation of low-carbon pilot policies during the sample period, PSM method, special industries, and standard errors clustered at the city level, the conclusion remains valid. The heterogeneity analysis indicates that for non-state-owned enterprises, high-pollution firms, those with lower financing constraints, and firms with lower production efficiency, market-incentive environmental regulatory policies have a stronger cost-reduction and efficiency-enhancement effect on businesses.

Our study makes several contributions to the literature. Firstly, we expand on previous research by focusing on the impact of the ERP on the operating costs of enterprises. Extensive research in the past has primarily focused on environmental investments, which do not directly reflect the existential pressures faced by firms. However, environmental costs encompass not only the expenses related to corporate environmental investments but also include the costs associated with environmental governance. This provides a more comprehensive consideration of the expenditures incurred by businesses. Second, we examine the impact of market-incentive ERP on the financial performance of enterprises, specifically whether companies can achieve a win-win situation of environmental protection and economic benefits under the pressure of market-incentive ERP. Third, we provide potential evidence for how enterprises can achieve high-quality development that integrates environmental protection with cost reduction and efficiency enhancement. Our findings reveal that green innovation is a crucial mechanism through which market-incentive environmental regulations exert their effects.

The rest of this article is organized as follows: Section 2 presents the literature review and hypothesis development. Section 3 introduces the samples, variables, and empirical models. Section 4 presents the empirical results and robustness tests. Section 5 explores underlying channels and heterogeneity analysis. Sections 6–9 cover the Discussion, Conclusion, Managerial Implications, and Practical Implications, respectively.

## 2. Literature review and theoretical analysis

### 2.1. Literature review of ERP

Generally, based on the classification method of the Organization for Economic Cooperation and Development (OECD), ERP are usually divided into two types: order-control ERP and market-incentive ERP [15].

Order-control ERP prevents the pollution behavior of enterprises through environmental legislation [16]. And in addition to clear legislative provisions, environmental enforcement agencies need to strictly enforce the law in actual implementation [17]. Generally, the order-control ERP is seen as the most effective means of environmental regulation [18]. However, some studies show that the government should further improve the system of environmental protection laws and regulations, increase the investment in environmental law enforcement, and improve the initiative of environmental law enforcement [19].

Different from Order-control ERP, market-incentive ERP operates through market mechanisms [17]. These systems can promote the optimal reallocation of resources among enterprises and rely on inter-enterprise effects to improve the aggregate productivity of industries [20]. At the same time, it can reduce the cost of public participation in environmental protection, and improve their interest in participating in environmental protection activities. Many studies have shown that market-incentive ERP systems should be further improved, by establishing more flexible emission charges and a stricter emission punishment system, to promote the further implementation of the emission trading mechanism [21].

### 2.2. Literature review of ERP’s economic outcomes

The influence of market-incentive ERP on both macro and micro economies has been a hot topic in this field [22–24].

The impact of market-incentive ERP on the economic structure is also a crucial area of research. From a macro perspective, a substantial body of research has affirmed the positive impact of market-incentive ERP on environmental improvement [25, 26]. For instance, the implementation of market-incentive ERP in some countries has not only reduced environmental pollution but also fostered the development of clean technologies and renewable energy industries [27, 28]. However, this process can also lead to an increase in emission reduction costs [29]. In the context of China, environmental regulatory policies may lead to increased compliance costs, thereby posing a barrier to high-quality economic development [30].

From a micro perspective, the impact of market-incentive ERP on enterprises is divided into environmental and economic effects. On the one hand, market-incentive ERP leads to emission reduction by enterprises [31–33], reduces pollution [34], promotes green innovation [35], and enhances green total factor productivity (TFP) [36]. The European Union’s Emissions Trading Scheme has demonstrated its ability to effectively reduce carbon emissions [33]. However, this reduction does not necessarily translate into direct economic benefits. On the other hand, some studies have examined the economic effects of ERP on enterprises [37, 38]. These studies find that the intensity of environmental regulations not only improves environmental quality but also fosters innovations that reduce operational costs, thereby enhancing firm performance. Unfortunately, existing literature has not provided accurate answers regarding the impact of market-incentive ERP, particularly CTP, on firms’ environmental costs and economic burdens.

### 2.3. Theoretical analysis

CTP can enhance environmental performance for businesses while potentially reducing environmental costs through the following avenues:

Firstly, market-incentive ERP aim to motivate enterprises to enhance their environmental performance through market mechanisms rather than solely relying on traditional order-and-control approaches [23]. Such market-oriented strategies may encourage companies to proactively seek methods to reduce environmental costs. For instance, the CTP incentivizes enterprises to reduce emissions by setting carbon emission quotas. When a company’s carbon emissions fall below their allocated quota, they can sell the excess in the market, thereby deriving economic benefits [39]. Therefore, this approach not only contributes to environmental protection but also provides an economic impetus for emission reduction.

Secondly, according to the Porter Hypothesis, environmental regulations can stimulate innovation within enterprises, thereby enhancing their long-term competitiveness [11]. Porter and Van der Linde (1995) noted that appropriate environmental regulations can prompt businesses to develop more efficient and cleaner technologies and processes, leading to cost savings and product innovation [11]. Such innovations not only reduce environmental costs but may also enhance a company’s market competitiveness.

Furthermore, market-incentive ERP, particularly the CTP, may achieve their objectives through green innovation, a key mechanism that links these policies with the reduction of environmental costs. ERP creates a market environment where environmental costs are internalized, compelling companies to innovate in green technologies and processes to maintain competitiveness [40, 41]. Companies that develop and implement green innovations can more effectively reduce their carbon footprint, thus lowering their emission quotas [42, 43]. This, in turn, potentially allows them to profit from selling surplus quotas, providing an incentive for enterprises to reduce environmental costs. Finally, market-incentive ERP elevate corporate awareness of environmental costs, steering businesses towards more sustainable business models [44]. This shift might involve redesigning existing production processes and products to minimize environmental impact and enhance resource efficiency. For instance, studies have shown that by adopting environmentally friendly production methods and energy-efficient technologies, companies can reduce energy consumption and waste generation, thereby decreasing environmental costs.

In summary, market-incentive ERP, particularly CTP, impact corporate environmental costs in multifaceted ways. This includes not only direct economic incentives but also indirect effects through promoting technological innovation and transforming business models to lower environmental costs.

## 3. Method and data

### 3.1. Data

Our sample consists of Chinese A-share-listed manufacturing enterprises between 2008 and 2020. The manufacturing sector classification is based on the 2012 Industry Classification List of the China Securities Regulatory Commission (CSRC). We obtained all the variables related to listed companies from the China Securities Market and Accounting Research (CSMAR) database. To screen the observations, we implemented the following process: (1) we excluded observations with missing data or only one date, (2) we removed corporations whose stock is labelled as “ST”, and (3) all continuous variables were winsorized at the 1%–99% levels. Ultimately, we formed a panel data set of 21,780 firm-year observations from 2,587 listed firms.

### 3.2. DID model

Since 2013, China has implemented the CTP in eight regions, namely Beijing, Shanghai, Tianjin, Hubei, Guangdong, Shenzhen, Chongqing, and Fujian which provides a quasi-natural experiment for this paper. To address endogeneity issues, we select listed manufacturing companies in these eight CTP regions as the experimental group and listed manufacturing companies in non-CTP regions as the control group.

To investigate the impact of market-incentive ERP on corporate environmental cost, we employ a DID model as follows, aiming to test the effect of the CTP on corporate environmental costs. Firstly, in line with the methodologies used by Qi et al. [45] and Liu et al. [46], we identify 2013 as the initiation year of CTP in seven regions, with the exception of Fujian, where CTP commenced in 2017. Secondly, to mitigate potential confounding factors in our regression analysis, we incorporate a set of firm-level control variables [47]. Lastly, we control for fixed effects at the individual, industry, and annual levels. The specific model is as follows:

$$Envi_{cost_{i,t}}=\alpha +\beta CTP_{i,t}+\gamma Controls_{i,t}+\mu_{i}+\theta_{t}+\varepsilon_{i,t}$$ (1)

In Eq (1), *Envi_cost* is the dependent variable, and *Envi_cost* represents the necessary expenditure related to the environmental protection of corporate *i* in year *t*. CTP is a proxy variable for carbon trading policy, and CTP is the key independent variable in our estimate. The coefficient and significance of *β* represent the impact of market-incentive ERP on corporate environmental expenditure. If *β* is negative and passes the significant test, it means that market-incentive ERP can significantly reduce corporate environmental cost. *Controls* represent a series of control variables, and *u*_*i*_ and *θ*_*t*_ represent firm fixed effects and time fixed effects, respectively.

### 3.3. Parallel trend test model

To ensure that our results are attributable to the treatment itself and not to other unobserved factors, we conducted parallel trend tests. This process helps confirm that, in the absence of intervention, the treatment and control groups exhibit similar trends. Following the approach taken by Jacobson, LaLonde [48] and Qiu [49] we use the parallel trend test model, which is presented as follows:

$$Envi_{cost_{i,t}}=\alpha +\sum_{\tau =-6,\tau \neq -1}^{6}\beta_{\tau }CTP_{i}\times I_{t}\left(t=\tau \right)+\gamma Controls_{i,t}+\mu_{i}+\theta_{t}+\varepsilon_{i,t}$$ (2)

### 3.4. Variable selection

#### 3.4.1. Dependent variable

Environmental cost: we follow the approach used in previous research on this topic [47, 50] and define the environmental cost of firms as the cost of environmental protection activities such as energy conservation, emission reduction, pollution prevention and control. According to the environmental expenditure list of listed companies collected by CSMAR, the corporate expenditures on environmental protection includes fees for sewage, solid waste, and dust treatment. These fees reflect the costs and expenditures necessary for companies to attain environmental standards. Therefore, we use the ratio of environmental costs to companies’ total assets as Envi_cost. And the data of corporate expenditures on environmental protection is collected from the "Construction in Progress" portion of corporate annual reports.

#### 3.4.2. Independent variable

CTP: Adhering to the approaches established by Qi et al. [45] and Liu et al. [46], we recognize 2013 as the starting year for the implementation of CTP across seven regions, while Fujian is an exception, having initiated CTP in 2017. CTP equals 1 if the company is registered in a province where the policy has been implemented, and 0 otherwise.

#### 3.4.3. Mediating variable

Green innovation: Green innovation involves adopting new technologies, methodologies, or products that reduce environmental pollution and optimize energy resource use [51]. Drawing on previous research [52], we use the annual number of green patent applications as a proxy for assessing corporate green innovation. These patents are classified based on the International Patent Classification Green Inventory, established by the World Intellectual Property Organization (WIPO) in 2010. We obtain the data on corporate green patents from the Chinese Research Data Services Platform (CNRDS) database.

#### 3.4.4 Control variables

Drawing on prior studies in this field [47, 50], we selected several control variables known to influence environmental costs. Firstly, we controlled for a range of fundamental corporate characteristics, specifically including corporate size (Size), financial leverage (Lev), return on assets (ROA), corporate age (FirmAge), and the turnover of total capital (ATO). Secondly, we incorporated control variables related to corporate governance, which encompass whether the Chairman and CEO roles are unified (Dual), the shareholding of the largest shareholder (Top1), and management fees (Mfee).

Table 1 provides the definitions of the variables and the data sources, while Table 2 reports the descriptive statistics of these variables.

**Table 1:** Variable define and data sources.

Variable	Variable Measure	Data sources
Envi_cost	The ratio of environmental costs to companies’ total assets	Corporate’s annual reports
CTP	CTP equals 1 if the company is registered in a province which CTP has been implemented; otherwise, 0	Direct assign
Size	The natural logarithm of the total assets at the end of the year	CSMAR
Lev	Total debt divided by total assets	CSMAR
ROA	Net profit/total assets	CSMAR
Dual	Dual = 1 if Chairman and CEO are the same person and 0 otherwise.	CSMAR
FirmAge	The natural logarithm of year of establishment	CSMAR
Mfee	Administrative expenses /operating income	CSMAR
Top1	shareholding of the largest shareholder	CSMAR
ATO	operating income/Total average assets	CSMAR
Green_innov1	Ln(number of green invention patents granted +1)	CNRDS
Green_innov2	Ln(number of green patents granted +1)	CNRDS
nSOE	nSOE = 1 if non-state owned / nSOE = 0 if state owned	CSMAR
KP	KP = 1 if the firm is a key polluting enterprise	CSMAR

**Table 2:** Descriptive statistics.

Variable	N	Mean	p50	SD	Min	Max
Envi_cost	21,780	0.022	0	0.132	0	1.081
CTP	21,780	0.255	0	0.436	0	1.000
Size	21,780	21.85	21.71	1.171	19.410	25.34
Lev	21,780	0.406	0.392	0.210	0.049	1.006
ROA	21,780	0.043	0.042	0.071	-0.267	0.235
Dual	21,780	0.301	0.000	0.459	0.000	1.000
FirmAge	21,780	2.781	2.833	0.376	1.386	3.466
Mfee	21,780	0.094	0.077	0.076	0.011	0.539
Top1	21,780	0.339	0.319	0.141	0.090	0.716
ATO	21,780	0.679	0.598	0.396	0.084	2.386
Green_innov1	21,780	0.128	0	0.422	0	2.398
Green_innov2	21,780	0.180	0	0.541	0	2.944
nSOE	21,780	0.704	1	0.456	0	1

## 4. Results

### 4.1. Baseline result

The baseline results are presented in Table 3. The first column (1) reports the results with only the dependent and control variables. The second column (2) includes industry and year fixed effects, and the third column (3) includes year and firm fixed effects. Finally, column (4) controls for the industry, year, and firm fixed effects.

**Table 3:** Baseline result.

	(1)	(2)	(3)	(4)
	Envi_cost	Envi_cost	Envi_cost	Envi_cost
CTP	-0.011***	-0.012***	-0.017***	-0.016***
	(-3.291)	(-3.624)	(-3.614)	(-3.460)
Size	0.010***	0.008***	-0.006*	-0.006**
	(6.129)	(5.106)	(-1.934)	(-2.091)
Lev	-0.020***	-0.006	-0.026**	-0.025**
	(-2.762)	(-0.904)	(-2.247)	(-2.170)
ROA	0.019	0.043**	0.039**	0.038**
	(1.059)	(2.554)	(2.161)	(2.112)
Dual	-0.001	-0.002	0.002	0.003
	(-0.300)	(-0.626)	(0.881)	(0.962)
FirmAge	0.018***	0.005	-0.016	-0.015
	(5.208)	(1.288)	(-1.236)	(-1.173)
Mfee	-0.019	0.004	-0.018	-0.015
	(-1.324)	(0.268)	(-1.003)	(-0.819)
Top1	0.010	0.017	-0.011	-0.014
	(0.843)	(1.634)	(-0.625)	(-0.766)
ATO	0.010***	0.008**	0.006	0.006
	(2.688)	(2.036)	(0.978)	(1.016)
_cons	-0.251***	-0.168***	0.201***	0.205***
	(-6.983)	(-5.071)	(2.734)	(2.822)
Ind	No	YES	No	YES
Year	No	YES	YES	YES
Firm	No	No	YES	YES
N	21,780	21,780	21,780	21,780
R^2^	0.015	0.076	0.358	0.360

Note:

***, **, and * indicate statistical significance at the 1%, 5%, and 10% levels, respectively. Standard errors clustered at the firm level.

The coefficient of CTP is negative and significant at the 1% level in each of the four columns, indicating that CTP significantly reduces environmental costs at the firm level. As shown in column (4) of Table 3, the coefficient of CTP is -0.016, and it passes the 1% significance test. This suggests that the Chinese CTP significantly reduces corporate environmental costs. Our findings support our initial hypothesis that market-incentive ERP can improve the financial performance of enterprises. We conclude that under the pressure of market-incentive ERP, firms can achieve a win-win outcome of environmental protection and economic benefits. Furthermore, the ROA shows a significant positive correlation with environmental costs. Companies with a high ROA often place greater emphasis on corporate social responsibility and maintaining a positive reputation [53, 54]. In today’s society, the public is increasingly concerned about corporate environmental responsibility and actions. To uphold their reputation and avoid negative public perception, companies with a high ROA may be more inclined to bear environmental costs to demonstrate their commitment to environmental protection.

### 4.2. Test on parallel trend assumption

**Fig 1:**
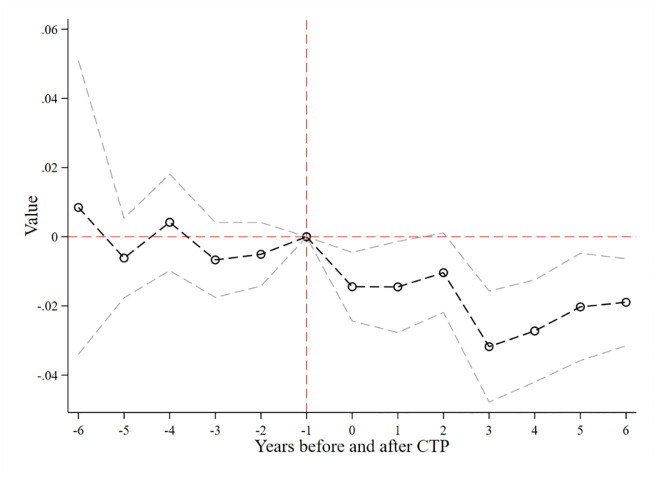
Test of parallel trend assumption. Note: For each coefficient, the 95% confidence interval is reported.

To ensure the validity of our DID estimates, a common trend between the treatment and control groups is necessary, as emphasized by Yang et al. [55]. Specifically, we assume that prior to the implementation of CTP, the trend in environmental costs was relatively stable. We perform OLS regressions on the dependent variables for the 6 years prior to and following the CTP year, respectively. We adhere to the approach of previous research and designate the year prior to the policy launch as the base year [56, 57]. The coefficients of environmental costs are not significantly different from zero in the 3 years prior to the CTP, indicating that the treatment and control groups satisfied the parallel trend assumption before the policy launch. Furthermore, the dynamic effect of the parallel trend test shows a rapid drop in environmental costs after the base year, with a turning point to negative and significant coefficients after the implementation of CTP (Fig 1). This policy effectiveness reaches its peak in the third year. These results support the validity of our DID estimates and suggest that CTP has a significant and lasting impact on corporate environmental costs.

### 4.3. Robust test

#### 4.3.1. Placebo test

**Fig 2:**
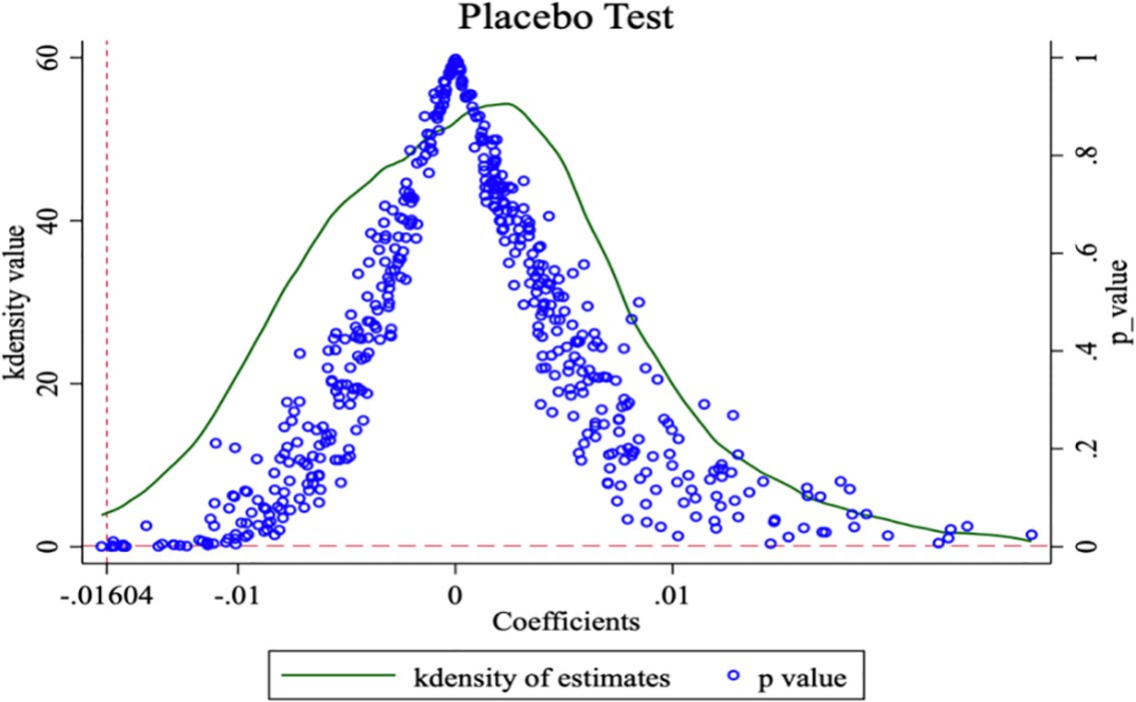
Placebo test.

To address concerns that the observed decline in environmental costs may be attributable to factors other than the CTP and that unobservable variables may affect the estimation results, we conducted a placebo test following the method of Chen et al. [26] and Yang et al. [55]. Specifically, we randomly selected eight provinces as CTP regions and randomized the policy time. After 500 iterations of random shocks, our result was an outlier from the mean value of the kernel density (Fig 2). We found that the mean value of the kernel density and P-values were both close to 0, indicating that there were no potential policy effects on environmental costs other than CTP. The results of this placebo test reinforce our primary finding, which is that the effects of CTP on environmental costs are significant and that other potential causes can be ruled out.

#### 4.3.2. Propensity score matching approach

We employ the propensity score-matching (PSM) approach to address the endogeneity problem arising from sample selection bias. Panel A displays the average treatment effect on treated of CTP. The results indicate that the environmental cost of corporations located in the carbon trading pilot region is approximately 0.0122 points lower than that of corporations in other regions (Table 4). Panel B presents the balance test of matching variables between the treated and control groups after the 1:2 neighbor matching method (Table 4). The results demonstrate that, following PSM, there are no significant differences between the Treated and Control groups.

**Table 4:** Propensity score matching approach.

Panel A: Average treatment effect on the treated							
Variable		ATT		Treated	Cnontrols	Treated:Controls	
CTP		-.0122*** (-4.87)		0.0163	0.0285	5,562:16,218	
Panel B: Balance test							
	Unmatched	Mean			%reduct	t-test	
Variable	Matched	Treated	Control	%bias	|bias|	t	P>|t|
Size	U	21.97	21.80	13.90	95.80	8.960	0.000
	M	21.97	21.96	0.600		0.300	0.765
Lev	U	0.388	0.413	-12.10	91.90	-7.680	0.000
	M	0.388	0.386	1		0.530	0.598
ROA	U	0.044	0.043	2	44.10	1.310	0.191
	M	0.044	0.045	-1.100		-0.590	0.555
Dual	U	0.383	0.273	23.50	89.20	15.45	0.000
	M	0.382	0.371	2.500		1.280	0.200
FirmAge	U	2.878	2.747	36.40	95.30	22.70	0.000
	M	2.878	2.884	-1.700		-1.020	0.309
Mfee	U	0.099	0.093	8.300	99.20	5.280	0.000
	M	0.099	0.099	0.100		0.030	0.974
Top1	U	0.329	0.343	-9.600	98.60	-6.270	0.000
	M	0.329	0.329	0.100		0.0700	0.943
ATO	U	0.658	0.687	-7.600	88.10	-4.780	0.000
	M	0.658	0.654	0.900		0.480	0.630

Note: Panel A shows the average treatment effect on treated of CTP. Panel B shows result of the balance test of matching variables between the treated and control groups.

#### 4.3.3. Exclude the distraction of low carbon pilot policy

Since 2011, China has implemented a low-carbon pilot policy in select cities. The resulting reduction in environmental costs for companies may be attributed to the impact of this policy. To ensure the robustness of our results and exclude any potential distractions caused by the low-carbon pilot policies, we conducted an additional analysis that removed all firms located in the low-carbon pilot cities. The results, presented in column (1) of Table 5, show that the coefficient of CTP remains negative and passes the 1% significance test.

**Table 5:** Robustness tests.

	(1)	(2)	(3)	(4)	(5)	(6)
	Envi_cost	Envi_cost	Envi_cost	Envi_cost	Envi_cost	Envi_cost
CTP	-0.019***	-0.014***	-0.020***	-0.016***	-0.016***	-0.015***
	(-2.738)	(-3.092)	(-4.080)	(-3.077)	(-3.435)	(-2.626)
GovEnv					0.001	
					(0.162)	
Anal						0.001
						(0.499)
Size	-0.008*	-0.011***	-0.007**	-0.006**	-0.006**	-0.008**
	(-1.887)	(-2.913)	(-2.306)	(-2.164)	(-2.092)	(-2.004)
Lev	-0.017	-0.019	-0.023*	-0.025**	-0.025**	-0.034***
	(-0.949)	(-1.370)	(-1.794)	(-2.194)	(-2.168)	(-2.616)
ROA	0.042	0.075***	0.042**	0.038**	0.038**	0.048
	(1.476)	(2.766)	(2.145)	(2.278)	(2.115)	(1.470)
Dual	0.001	0.006*	0.003	0.003	0.003	0.003
	(0.263)	(1.663)	(1.019)	(0.922)	(0.969)	(0.756)
FirmAge	-0.003	-0.018	-0.011	-0.015	-0.015	-0.017
	(-0.138)	(-1.065)	(-0.783)	(-1.130)	(-1.170)	(-1.150)
Mfee	-0.019	-0.013	-0.016	-0.015	-0.015	-0.044
	(-0.817)	(-0.808)	(-0.778)	(-0.814)	(-0.821)	(-1.019)
Top1	0.012	0.001	-0.004	-0.014	-0.014	-0.009
	(0.409)	(0.053)	(-0.206)	(-0.674)	(-0.769)	(-0.437)
ATO	-0.001	-0.003	0.007	0.006	0.006	0.009
	(-0.069)	(-0.368)	(1.051)	(0.995)	(1.015)	(1.188)
_cons	0.207*	0.305***	0.218***	0.205***	0.202***	0.253***
	(1.897)	(3.410)	(2.725)	(3.102)	(2.676)	(2.610)
Ind	YES	YES	YES	YES	YES	YES
Year	YES	YES	YES	YES	YES	YES
Firm	YES	YES	YES	YES	YES	YES
N	10,473	14,616	18,778	21,780	21780	15282
R2	0.337	0.361	0.363	0.360	0.360	0.371

Note:

***, **, and * indicate statistical significance at the 1%, 5%, and 10% levels, respectively. Standard errors in columns (1)-(3) are clustered at the firm level and in column (4) is clustered at the city level.

#### 4.3.4. Exclude the effect of the national carbon trading market on the electricity industry

Beginning in 2017, China launched a national carbon trading market for the electricity industry. This may have an impact on our results, so we used a sample spanning from 2008 to 2017 to exclude the effect of the national carbon trading market on the electricity industry. Column (2) presents the results of this analysis, showing that the coefficient of CTP remains negative and significant (Table 5).

#### 4.3.5. Exclude companies in municipalities directly under the central government

China has four municipalities directly under the central government (Beijing, Chongqing, Shanghai and Tianjin) and they have unique status and policies. How companies operate in these regions may differ from other areas. In this paper, however, all four municipalities are CTP pilot regions, which may affect the regression results due to the specificity of the municipalities rather than the CTP pilot. To address this concern, we conducted a robustness test by removing listed companies in these municipalities. Column (3) presents the results of this sub-sample analysis, which shows that the negative and significant coefficient of CTP remains after removing the listed manufacturing companies in the municipalities (Table 5).

#### 4.3.6. Standard errors were clustered at the city level

Our previous baseline estimates clustered standard errors at the firm level. To further ensure the robustness of our results, we changed the standard error to the city level. Our analysis in column (4) shows that the coefficient of CTP remains negative and significant when standard errors are clustered at the city level. Thus, we conclude that our findings are robust even after conducting a series of robustness tests (Table 5).

#### 4.3.7. Exclude the effect of government regulatory pressure and public opinion supervision pressure

We further considered the impact of government regulatory pressure and public opinion supervision pressure on environmental costs. First, we utilized Python to conduct word segmentation on government work reports and calculated the frequency of keywords related to environmental regulations in provincial government annual work reports as a proxy for local government’s environmental pressure [58]. Second, we used the number of analyses and attention received by enterprises as a proxy for external supervision pressure [59]. Column (5) and (6) respectively indicate that the impact of CTP on corporate environmental costs remains significant even after excluding the effect of government regulatory pressure and public opinion supervision pressure.

## 5. Additional analysis

### 5.1. Mechanism analysis

This paper empirically tested the effect of market-incentive ERP on a firm’s environmental costs, finding a significant reduction. This section explores the possible mechanisms through which CTP influences corporate environmental costs. Green innovation has been recognized as a crucial source of sustainable development [60–63]. According to Chang [52] and Tang, Walsh [64], green innovation can reduce pollution, minimize waste, and lower a company’s environmental expenditure. Green innovation can lead to optimization and improvement of production processes, thereby increasing production efficiency and reducing environmental-related production costs for businesses. Additionally, green innovation involves the adoption of more advanced pollution control equipment and processes, thereby lowering the costs associated with treating emissions and pollutants.

As mentioned previously, CTP provides enterprises with flexible options for making decisions on the direction of their environmental expenditure: (1) maintaining the current technology level and paying the necessary environmental costs to meet government standards, or (2) reducing environmental costs through green technology innovation. In this case, green innovation reduces environmental costs and may yield market benefits. Therefore, we hypothesize that green innovation provides the pathway for CTP to influence a firm’s environmental costs.

We followed Liu and Li [65] to test this mechanism and obtained corporate green patent data from the Chinese Research Data Services Platform (CNRDS) database. Table 6 shows the regression results of the CTP on green innovation. In column (1) and (2), the dependent variable is the number of green invention patents granted; in column (3) and (4), it is the number of green patents granted. As reported in Table 6, the CTP significantly promotes corporate green innovation.

**Table 6:** Carbon trading policy and corporate green innovation.

	(1)	(2)	(3)	(4)
	Green_innov1		Green_innov2	
CTP	0.053***	0.050***	0.056***	0.052**
	(2.863)	(2.712)	(2.493)	(2.329)
Size		0.073***		0.096***
		(7.736)		(8.064)
Lev		-0.018		-0.038
		(-0.610)		(-1.063)
ROA		0.002		-0.041
		(0.053)		(-0.706)
Dual		0.005		0.002
		(0.570)		(0.135)
FirmAge		-0.036		-0.019
		(-0.702)		(-0.297)
Mfee		0.107*		0.101
		(1.771)		(1.345)
Top1		0.028		0.010
		(0.418)		(0.120)
ATO		0.010		0.016
		(0.567)		(0.763)
_cons	0.114***	-1.392***	0.166***	-1.880***
	(24.104)	(-5.486)	(28.676)	(-5.969)
Ind	YES	YES	YES	YES
Year	YES	YES	YES	YES
Firm	YES	YES	YES	YES
N	21,780	21,780	21,780	21,780
R2	0.562	0.567	0.586	0.590

Note:

***, **, and * indicate statistical significance at the 1%, 5%, and 10% levels, respectively. Standard errors clustered at the firm level.

### 5.2. Heterogeneous analysis

In this section, we conducted a heterogeneity analysis based on four factors: the ownership structure of the enterprise, whether it is classified as a key polluting enterprise (KP = 1 if the enterprise is a key polluting enterprise, and 0 otherwise.), whether it is a high financial constraint enterprise (KZ_index = 1 if a firm’s KZ index is greater than the median value, and 0 otherwise), and whether it is a high total factor productivity enterprise (TFP = 1 if a firm’s TFP value is greater than the median value, and 0 otherwise).


$$\begin{array}{ll} Envi_{cost_{i,t}} & =\gamma_{0}+\gamma_{1}CTP_{i,t}*nSOE_{i,t}+\gamma_{2}nSOE_{i,t}+\gamma_{3}CTP_{i,t}+\gamma Controls_{i,t}\\ & +\mu_{i}+\theta_{t}+\varepsilon_{i,t} \end{array}$$ (3)

$$\begin{array}{ll} Envi_{cost_{i,t}} & =\beta_{0}+\beta_{1}CTP_{i,t}*KP_{i,t}+\beta_{2}KP_{i,t}+\beta_{3}CTP_{i,t}+\beta Controls_{i,t}+\mu_{i}\\ & +\theta_{t}+\varepsilon_{i,t} \end{array}$$ (4)

$$\begin{array}{ll} Envi_{cost_{i,t}} & =\varphi_{0}+\varphi_{1}CTP_{i,t}*KZ\_Index_{i,t}+\varphi_{2}KZ\_Index_{i,t}+\varphi_{3}CTP_{i,t}\\ & +\varphi Controls_{i,t}+\mu_{i}+\theta_{t}+\varepsilon_{i,t} \end{array}$$ (5)

$$\begin{array}{ll} Envi_{cost_{i,t}} & =\omega_{0}+\omega_{1}CTP_{i,t}*TFP_{i,t}+\omega_{2}TFP_{i,t}+\omega_{3}CTP_{i,t}+\omega Controls_{i,t}\\ & +\mu_{i}+\theta_{t}+\varepsilon_{i,t} \end{array}$$ (6)

**Fig 3:**
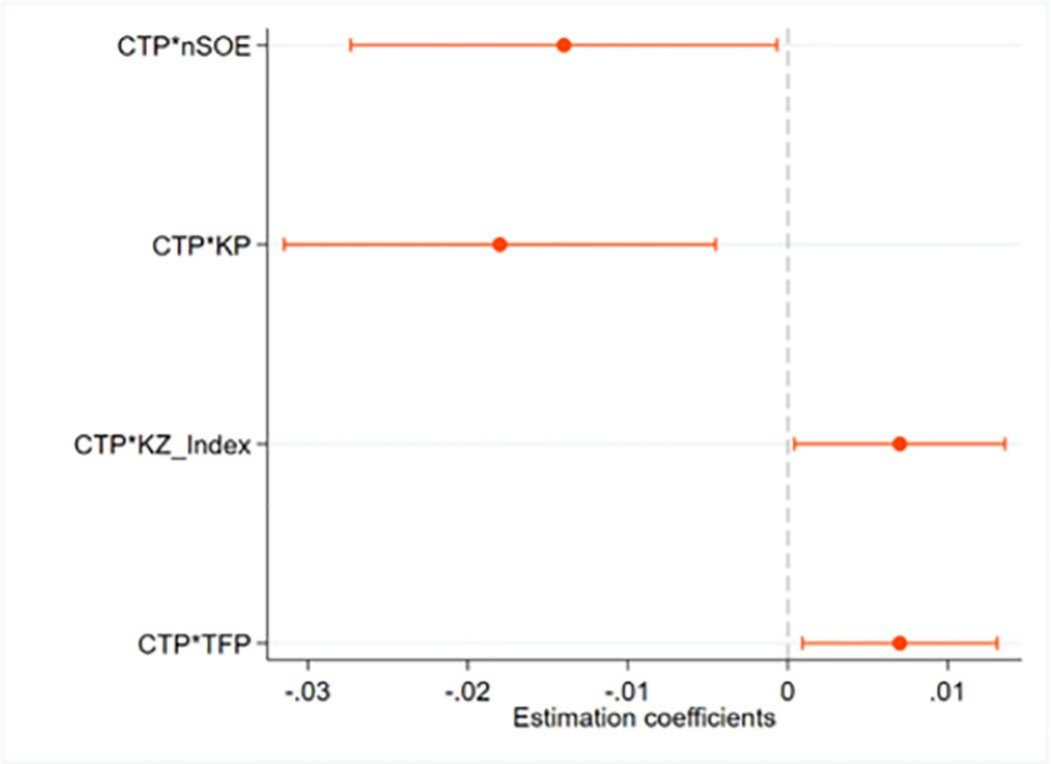
Heterogeneous analysis. Note: We report the results of models (3) to (6), where dots and lines represent the estimated coefficients and the 90% confidence intervals of the interaction terms between CTP and heterogeneous variables (the ownership structure of the enterprise; whether it is classified as a key polluting enterprise; whether it is a high financial constraint enterprise; and whether it is a high total factor productivity enterprise).

First, State-owned enterprises (SOEs) and non-state-owned enterprises (non-SOEs) both hold significant positions in the Chinese economy [66]. While non-SOEs prioritize corporate performance and economic returns, SOEs are also expected to perform certain government functions [67]. Consequently, non-SOEs are typically more sensitive to policy changes and cost fluctuations, making them more susceptible to the impact of CTP on environmental costs. As shown in Fig 3, the coefficient of the interaction between CTP and non-SOEs is significantly negative, indicating that the negative relationship between CTP and corporate environmental costs is stronger for non-SOEs, highlighting the potential of CTP to reduce the environmental costs of these firms. From the perspective of eco-innovation analysis, carbon emission trading policies have a more significant impact on reducing environmental costs for non-SOEs. On the one hand, non-SOEs often operate in highly competitive markets [68], which exert significant pressure on them to reduce costs and enhance competitiveness. CTP provide these enterprises with opportunities to participate in green innovation and obtain carbon emission allowances, further incentivizing them to lower environmental costs. On the other hand, non-SOEs are typically more flexible and market vitality [69], making them better equipped to adapt to carbon emission trading policies. These enterprises are more inclined to adopt eco-innovative technologies and management measures to lower carbon emissions and reduce environmental costs.

Second, the coefficient of the interaction between CTP and KP is significantly negative, which reveals that key polluting enterprises in CTP areas have a greater incentive to reduce their environmental costs. These enterprises generally have much higher environmental costs than other firms, making them more likely to invest in green technology innovation to reduce their environmental expenses. Therefore, CTP is expected to impact these companies more substantially.

Third, we constructed a dummy variable based on firms’ financing constraints to examine the heterogeneous impact of financing constraints on firms. The level of financing constraints a firm faces is measured using the KZ index [70], with a higher KZ index indicating greater financing constraints. As shown in Fig 3, the coefficient of the interaction between CTP and KZ_Index is positive and significant at least at the 10% level. The impact of CTP on corporate environmental costs is influenced by the level of the firm’s financing constraints, with firms facing higher financing constraints exhibiting a lower negative impact of CTP on environmental costs compared to firms with lower financing constraints. This may be due to firms with high financial constraints having limited capacity to obtain external funding, which restricts their ability to make necessary investments to meet environmental regulations and implement green technologies. Consequently, even with the incentives provided by the CTP, these enterprises struggle to secure sufficient funds to implement measures that would reduce environmental costs.

Finally, we further analyze the heterogeneous impact of firm efficiency. We choose total factor productivity as the metric to measure firm efficiency. Total factor productivity is an indicator that measures a firm’s production efficiency considering all production factors, such as labor and capital. It reflects how effectively a firm can transform these inputs into outputs. The impact of the CTP on corporate environmental costs varies when firms have different levels of TFP. As shown in Fig 3, the interaction term between CTP and TFP is significantly positive, indicating that the CTP’s role in reducing environmental costs is more pronounced in firms with lower firm efficiency. From the perspective of diminishing marginal utility, firms with high firm efficiency typically have already achieved higher efficiency in resource utilization, which implies that they may have optimized their environmental management and energy usage. In contrast, firms with low firm efficiency may have more room for improvement in these areas, thus the stimulative effect of CTP could be more evident in these firms.

## 6. Concluding remarks

### 6.1. Conclusions

This article aims to investigate the casual effect of market-incentive ERP on the enterprises’ environmental cost. To address the endogenous problem, we take the implementation of CTP as a quasi-natural experiment and employ a DID model to estimate the real effect. Specifically, a sample of China’s A-share listed manufacturing firms from 2008 to 2020 is used to conduct a quasi-natural experiment. Our findings are as follows. First, market-incentive ERP can effectively reduce the environmental cost of enterprises. Second, the impact is particularly pronounced for non-SOEs, key polluting firms, firms with lower financial constraints, and firms with lower total production efficiency. Third, this effect is primarily driven by enterprises’ increased investment in eco-innovation, particularly green innovation.

However, there are still some limitations. First, our findings are based on the samples of listed companies. Because of the data limitation, we can only get the latest samples by using Chinese listed firms. If possible, the future research can collect the small and medium-sized enterprises as samples. Second, we include all listed manufacturing companies in our sample, rather than focusing solely on those listed manufacturing enterprises identified as polluters. Because of the availability of polluting enterprises data, we can only know whether a firm is a key polluting one or not. And the number of key polluting enterprises is very small. We can only get the average effect of market-incentive environment regulation policy on enterprise environmental cost.

### 6.2. Discussions, managerial implications and practical implications

#### 6.2.1. Discussions

Overall, our results from the difference-in difference model are consistent with the theoretical analysis, which means that market-incentive environment policy can reduce enterprises’ burden caused by environment protection. This article is different from the existing literature by analyzing the effect of environmental regulation on environmental expenditure. In addition, our study provides compelling evidence that market-incentive ERP can lead to a "double dividend" for pollution-intensive firms, promoting both environmental protection and economic performance. This insight is of great value to policymakers in emerging markets seeking to achieve sustainable economic development in the long term.

#### 6.2.2. Managerial implications

Increasing public awareness of environmental protection and low-carbon development is important. The government and enterprises can work together to promote environmental education and encourage the public to adopt environmentally friendly lifestyles. In this way, a low-carbon lifestyle can become a social norm, and sustainable development can be achieved in the long run.

#### 6.2.3. Practical implications

In addition to the managerial implications mentioned above, there are other measures that the Chinese government and enterprises can take to promote sustainable economic development. First, the government can strengthen environmental law enforcement and supervision. Implementing environmental regulations and policies can effectively constrain the pollution behavior of enterprises and enhance their awareness of environmental protection. Additionally, the government can establish a green tax system to encourage enterprises to reduce their carbon emissions. Second, enterprises can take proactive measures to reduce their carbon footprint. For example, they can adopt cleaner production processes, use renewable energy sources, and develop environmentally friendly products. Enterprises can improve their competitive advantage and enhance their brand reputation by doing so.
